# Access to Healthcare Following Serious Injury: Perspectives of Allied Health Professionals in Urban and Regional Settings

**DOI:** 10.3390/ijerph18031230

**Published:** 2021-01-29

**Authors:** Jemma Keeves, Sandra C. Braaf, Christina L. Ekegren, Ben Beck, Belinda J. Gabbe

**Affiliations:** 1 Department of Epidemiology and Preventive Medicine, Monash University, Melbourne 3004, Australia; sandra.braaf@monash.edu (S.C.B.); christina.ekegren@monash.edu (C.L.E.); Ben.Beck@monash.edu (B.B.); belinda.gabbe@monash.edu (B.J.G.); 2 Department of Physiotherapy, Epworth Hospital, Melbourne 3122, Australia

**Keywords:** health services accessibility, delivery of healthcare, geography, allied health, wounds and injuries, qualitative research, rural population, urban population

## Abstract

Barriers to accessing healthcare exist following serious injury. These issues are not well understood and may have dire consequences for healthcare utilisation and patients’ long-term recovery. The aim of this qualitative study was to explore factors perceived by allied health professionals to affect access to healthcare beyond hospital discharge for people with serious injuries in urban and regional Victoria, Australia. Twenty-five semi-structured interviews were conducted with community-based allied health professionals involved in post-discharge care for people following serious injury across different urban and regional areas. Interview transcripts were analysed using thematic analysis. Many allied health professionals perceived that complex funding systems and health services restrict access in both urban and regional areas. Limited availability of necessary health professionals was consistently reported, which particularly restricted access to mental healthcare. Access to healthcare was also felt to be hindered by a reliance on others for transportation, costs, emotional stress and often lengthy time of travel. Across urban and regional areas, a number of factors limit access to healthcare. Better understanding of health service delivery models and areas for change, including the use of technology and telehealth, may improve equitable access to healthcare.

## 1. Introduction

Equitable access to healthcare is a public health priority area [1]. Limited access and availability of adequate health services following serious injury have been reported as a potential contributing factor to poor long-term health outcomes [2,3,4]. With improvements in acute trauma care, there is a growing number of people living with the burden of serious injury who require ongoing healthcare for years following injury [5,6].

The centralisation of specialised trauma care in urban areas may negatively impact healthcare access and utilisation for people residing in regional areas beyond hospital discharge [4,7]. Previous work exploring the perspectives of health professionals managing people with serious injuries has found that there were a number of challenges in providing optimal care to patients across regional and urban areas [8]. Access to healthcare is not an easily defined concept. Multiple factors, relating to the accessibility of a health service and to the healthcare-seeking individual, can impact healthcare utilisation [9]. Known factors impacting access to care following serious traumatic injury include, but are not limited to, the geographic location of services, individual travel needs and coordination of care from acute to post-discharge healthcare [3,4,7,8,10].

The management of people with serious injuries in the community is complex and often requires a wide variety of allied health professionals (AHPs) to aid rehabilitation and community reintegration [11]. To maximise the potential for recovery of pre-injury function, employment and quality of life, it is essential that appropriate services are accessible and available for those with long-term functional limitations [5,12]. Additionally, the perspectives of AHPs have been used to offer recommendations to inform and improve post-discharge care for people with serious injuries [10,13].

The aim of this qualitative study was to explore factors perceived by allied health professionals to affect access to post-discharge healthcare for people with serious injuries in urban and regional areas of Victoria. Allied health professionals based in major cities were classified as urban, and those practicing in inner regional and outer regional areas of Victoria, as regional [14].

## 2. Materials and Methods

### 2.1. Setting

Victoria is a state of Australia with a population of 6.46 million people [15], 76% residing in metropolitan areas, 23% in regional towns and <1% in areas considered to be “Remote” or “Very Remote” [15]. Seriously injured people are assessed and transported to appropriate acute care services based on pre-hospital triage and inter-hospital transfer guidelines. Serious injury includes spinal cord injury (SCI), traumatic brain injury (TBI), or orthopaedic injuries that required an emergency hospital admission [8]. There are two adult and one paediatric major trauma services in Victoria, all located in inner metropolitan Melbourne, which provide definitive care for 79% of major trauma cases in Victoria [16].

Funding for injury care in Australia varies depending on state-based legislation. In Victoria, there are two main injury insurance schemes, both no-fault third-party insurers, with one primarily covering transport-related injury and the other workplace injury. These injury insurance schemes provide compensation for medical expenses, rehabilitation and support services as well as financial assistance. For those not compensated by these schemes, care is funded by Medicare, Australia’s publicly funded universal healthcare system for all citizens and permanent residents, and/or private health insurance. In 2016, the National Disability Insurance Scheme (NDIS) was introduced at a federal level to provide individualised support for Australians under the age of 65 years, living with permanent and significant disability [17].

### 2.2. Recruitment Strategy

Purposive sampling was used to recruit a broad range of AHPs in different geographic regions with at least 12 months experience in caring for adults following serious injury after acute hospital discharge [18]. For the purpose of this study, an AHP was defined as a university-qualified health professional, not part of the medical, dental or nursing professions, with specialised expertise in preventing, diagnosing and treating a range of conditions [19]. This definition includes the professions of audiology, chiropractic, dietetics, exercise physiologists, occupational therapy, optometry, orthoptics, orthotics/prosthetics, osteopathy, physiotherapy, podiatry, psychology, social work, sonography and speech pathology [19]. Purposive sampling criteria were based on geographic area (according to Accessibility/Remoteness Index of Australia (ARIA) classifications [14]) and health profession. Urban AHPs were recruited from major cities and regional AHPs from inner regional and outer regional areas as per ARIA definitions [14]. Allied health professionals with experience in both urban and regional areas were considered as part of a state-wide group. More physiotherapists were recruited for this study as they make up the largest group of AHP in Australia and are more consistently involved in the long-term care of this population [20].

Participants were initially recruited via advertisements in newsletters of professional governing bodies, such as the Australian Physiotherapy Association, Occupational Therapy Australia and Services for Australian Rural and Remote Allied Health, relevant social media channels and snowball sampling. A limited number of participants from regional areas were recruited following the first three months of this study. As such, the recruitment strategy was expanded to include direct contact with health services via phone and email. Potential participants responded to the advertisement via email. Allied health professionals who expressed interest were emailed further information and the participant information sheet. A follow-up email was sent approximately one week later to clarify study details and organise the interview. Due to geographical limitations, interviews were completed by video- or teleconferencing, based on participant preference and audio recorded. The Monash University Human Research Ethics Committee (ID_12705) approved the project.

### 2.3. Data Collection

A single trained female, qualitative interviewer and physiotherapist (J.K.) conducted individual semi-structured interviews in English by video or teleconferencing, with only the participants and researchers present. Verbal consent was gained at the commencement of the interview. An interview guide was developed based on research aims and qualitative literature in injured populations. Questions were further informed by discussions with the project team based on their experience interviewing people with injuries. Questions about the availability of resources and services, interactions with people following serious injury and experiences with different funding models were also asked with additional prompting for more information if required (Appendix A). Interviews ranged from 45 to 75 min in duration. The sample size in this study was guided by the experience of the research team and analysing the data in relation to the goals of the research [21]. Data saturation was evident after the completion of 25 interviews and recruitment was then ceased.

### 2.4. Data Analysis

Interpretative phenomenological analysis (IPA) was used in this qualitative study to gain a detailed understanding of factors affecting access to healthcare for people with serious injuries in urban and regional areas as perceived by allied health professionals [22]. IPA is a useful methodology for investigating complex and unclear topics [22]. By examining the lived experience of each participant’s account, insights are gained about the meaning ascribed to the phenomena under study [22]. Analysts interpret the dataset without applying pre-existing theoretical preconceptions [22].

Thematic analysis using a reflexive approach was used, as described by Braun and Clarke [23,24]. This method was chosen due to its flexibility in application and focus on extracting meaning from data which is well aligned with the IPA methodological approach. NVivo 11 (QSR International Pty Ltd. Doncaster, Australia) was used for data management and coding which occurred concurrently with data collection. The initial five transcripts were double coded (J.K. and S.C.B.) to develop the coding framework and ensure coding reliability [25]. Inductive and iterative thematic analysis was used to generate initial themes from which a thematic framework was created, with ongoing revisions to ensure it was representative of the dataset. Themes and subthemes were finalised through ongoing author discussions until there was agreement. A number of strategies were implemented through the project to ensure rigour and trustworthiness of this qualitative research (Table 1).

## 3. Results

Twenty-five semi-structured interviews were completed with AHPs between December 2018 and April 2019 (urban, *n* = 8; regional, *n* = 14; state-wide (i.e., urban and regional), *n* = 3). This study included 13 physiotherapists (PTs), five occupational therapists (OTs), one social worker (SW), two neuropsychologists and four exercise physiologists (EPs) (Table 2). All participants who expressed interest in this study agreed to be interviewed.

**Figure 1:**
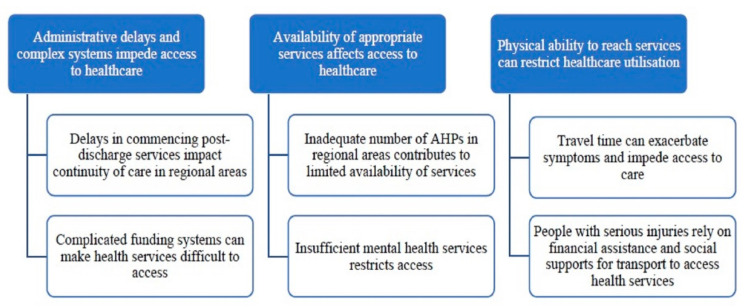
Thematic framework of key themes and subthemes.

Three key themes and multiple subthemes were identified through qualitative analysis (Figure 1). Differences between urban and rural experiences are described within the results. Participants’ study number, regionality, profession, gender and age range in years are reported to provide context for supporting quotes. An asterisk at the end of a quote indicates that further detail can be found in Appendix B.

### 3.1. Administrative Delays and Complex Systems Impede Access to Healthcare

#### 3.1.1. Delays in Commencing Post-Discharge Services Impact Continuity of Care in Regional Areas

In comparison to urban areas, people in regional areas reported lengthy delays in commencing post-discharge health services.

*“The clients I see in the country … they take months to get a team organised after discharge … In the city that would happen in a week.”*

ID 14_Statewide_Other_F_37-43

Delays were pimarily attributed to poor coordination and transparency of information beween health services.

*“… unfortunately, the most common scenario is that the referral from the metro hospital hasn’t been activated … So they’ve been discharged from hospital, sent back to [regional town] and then no follow-up rehab has happened … we would see those guys potentially four, five, unfortunately sometimes six months post-accident.”*

* ID17_Regional_EP_M_30-36

As well as delays in commencing initial outpatient allied health services, AHPs reported that reviews by rehabilitation consultants were delayed for people in regional areas, with the perception that people living in regional areas *“kind of disappear off the system” [ID14]*.

#### 3.1.2. Complicated Funding Systems Can Make Health Services Difficult to Access

Both urban and regional AHPs referenced the challenges that they experienced in navigating different funding models and understanding what each system was able to fund. These challenges were raised as frustrations for both the AHP and the injured individual and often restricted access to services.

*“… every different agency is so different, and that’s the frustration. There are all different policies and procedures, and it always seems a lot harder to provide the services with [Injury_Insurer_1].”*

ID15_Urban_OT_F_30–36

On the other hand, there were elements of injury insurance systems such as pre-approval of services, which AHPs noted to be a significant facilitator for timely access to services.

*“Since [Injury_Insurer_2] changed their funding model to moving to a pre-approved approach has been really beneficial for patients and for the therapy team … We know we can put together a program and not be placing our organisation at financial risk, and putting the patient under extra stress to say we have to wait for things to be approved.”*

ID22_Regional_PT_M_37–43

Both urban and regional participants also noted that they were required to strongly advocate for their patients with funding bodies to access necessary services or equipment, creating an additional administrative burden associated with the management of people with serious injuries.

*“I think if those sorts of [NDIS] services aren’t flexible enough from a funding perspective to allow quick access to funds, and things like that, that you end up fighting tooth and nail just to get someone what they’re actually entitled to.”*

ID24_Regional_OT_F_37–43

### 3.2. Availability of Appropriate Services Affects Access to Healthcare

#### 3.2.1. Inadequate Number of AHPs in Regional Areas Contributes to Limited Availability of Services

Allied health professionals working in state-wide or regional health services consistently raised that a lack of AHPs contributed to limited availability of healthcare in regional areas. They noted that whilst necessary services were often available, the intensity of therapy able to be provided was far less than what people following serious injury would receive in urban areas.

*“… physios are booked out for weeks in advance, but someone needs intensive physiotherapy … obviously [physiotherapy] is an existing resource, you’re still always scraping for appointments in a timely manner. That goes for all allied health. The speech therapist has waiting lists, the dietetics have waiting lists. Sorry, everyone has waiting lists.”*

ID7_Regional_Other_F_30–36

Additionally, the importance of accessing AHPs with appropriate expertise for people with complex needs was highlighted as a challenge in regional areas.

*“… there’s not enough therapists in that town, or their therapists that don’t have the level of expertise that they might need for their really complex therapy needs.”*

* ID12_Statewide_PT_F_30–36

For people in regional areas, limited availability of medical specialists and longer waiting periods for consults were perceived to be a significant barrier to accessing timely care.

*“when you’re dealing with more complex patients there will be a lot of different [medical specialists] involved … which can be a little bit more challenging, because you’re going to have to wait til they travel out this way from a bigger hub, or patients have to travel a long way to get to them, and often wait a long time to see them.”*

ID2_Regional_EP_M_23–29

#### 3.2.2. Insufficient Mental Health Services Restricts Access

Allied health professionals from urban and regional areas consistently reported the importance of early emotional and psychology support to help people with serious injuries to maximise their long-term health outcomes.

*“… that’s a real key, that if clients were provided with the emotional support and the psychological support right from the word go, and that ability for them to be able to accept their injury, and work with the allied health professionals, then they would have much more positive outcomes.”*

* ID8_Regional_OT_F_44–51

In urban and regional areas, participants talked about the cost of psychology services being a barrier to access for non-compensable patients. They reported “a real gap with social work services…and even access to psychology”* [ID7]. For non-compensable people with injuries, accessing psychology, “has to be through their GP, getting onto a (mental health) care plan”* [ID6]. The limited number of psychology sessions available through mental healthcare plans was also insufficient to meet patient needs and not always financially accessible due to out-of-pocket expenses.

*“navigating (GP) mental health care plans … even the Medicare rebate in psychology is out of reach for a lot of people … then there is still a huge gap for each appointment. That’s not achievable for a lot of people.”*

* ID7_Regional_Other_F_30–36

Despite having funding for psychology, people receiving compensation for their injuries still experience a skills and service shortage relating to mental health support in regional areas.

*“And the country clients are really disjointed … there are no neuropsyches in the country. It’s crazy, they fund me 3½ hours, 4 h to drive each way to see a client because there’s no-one closer … it’s completely nuts.”*

ID14_Statewide_Other_F_37–43

### 3.3. Physical Ability to Reach Services Can Restrict Healthcare Utilisation

#### 3.3.1. Travel Time Can Exacerbate Symptoms and Impede Access to Healthcare

Participants in both urban and regional areas reported that travel time to services impacted accessibility as it commonly exacerbated pain, anxiety and fatigue.

*“A lot of clients talk about how hard [travelling to Melbourne] is, whether it’s because of pain … [the travel] brings up a lot of anxiety for them …”*

ID19_Urban_PT_F_30–36

Difficulty travelling for appointments was often in relation to people from outer metropolitan and regional areas being required to attend medical consultations at inner metropolitan facilities. This resulted in frustrations from people having “*travelled two hours each way, and seen the specialist for five minutes*” *[ID18]*. As a result, AHPs reported patients choosing not to attend reviews or were physically limited and unable to access services.

*“there’s problems when the patients go back to the acute hospitals, to the big trauma hospitals in the city, for reviews and plans … a lot of people from the country don’t like going up to the big city, or the big city hospitals, they find it really stressful and annoying.”*

* ID3_Urban_Other_F_37–43

The use of telehealth was raised as an opportunity to minimise unnecessary travel and improve accessibility for people in urban and regional areas. Whilst AHPs felt telehealth could be very beneficial for this clientele, they reported cultural resistance from medical specialists in urban hospitals as a barrier to the successful implementation of telehealth.

*“I think telehealth can play a much bigger part in [medical reviews] … But it’s really hard to get specialists to consider telehealth as an option.”*

ID19_Urban_PT_F_30–36

#### 3.3.2. People with Serious Injuries Rely on Financial Assistance and Social Support for Transport to Access Health Services

In addition to the exacerbation of symptoms, AHPs reported that the financial costs and logistics of a trip to the city were significant enough that some people were not able to attend medical review appointments. This made it difficult for therapists to progress rehabilitation.

*“A lot of our clients have to travel. And because a lot of them are waiting compensation claims, or awaiting trying to get on the [disability support pension]. Their finances are in a pretty poor state … a lot of them will not go to appointments, or not schedule appointments because of the costs of travelling to Melbourne to see those specialists.”*

ID4_Regional_OT_F_37–43

While financial reimbursements were available for people living in regional areas to help make the costs of travelling less burdensome, AHPs reported “*even navigating that financial reimbursement is a nightmare*” *[ID7]* and reimbursements were often insufficient to cover costs.

*“I’ve got elderly people catching public transport, which is really inappropriate… We’ve got people that just don’t go down to their follow-up appointments because they literally can’t get down there.”*

ID7_Regional_Other_F_30–36

With the introduction of the NDIS, participants reported that some of the financial assistance provided for aspects of their care was beneficial for those in urban and regional areas. However, if the patient was unable to self-fund items such as additional equipment or an appropriate vehicle that the NDIS would not cover, their access could still be restricted.

*“The NDIS pays for vehicle modifications, but for someone who requires a van to get around… sometimes that’s just too expensive for the participant to buy, so they have no way of accessing the local community … they’re very limited.”*

ID12_Statewide_PT_F_30–36

Across both urban and regional AHPs, there was widespread belief that people under compensable funding systems have better access to health services.

*“[*For compensable patients*] I think there’s more available services because by funding carers and trainers you can get access to community facilities that are setup, and the carer can provide the support in that facility, which a non-compensable patient struggles to access.”*

ID10_Urban_PT_F_51+

Following serious injury, many people are unable to drive for months due to medication effects, non-weightbearing status or functional limitations. As such, they rely heavily on family, friends, carers and taxis to assist with transportation, creating “*a real issue for a lot of people*” * *[ID12].* In urban areas, people receiving injury compensation “*tend to be not too restricted in … accessing the community because they have [funded] taxis, or funded carers …*” *[ID13]*.

Transportation was also an issue for people in urban areas if they did not have funded taxi transport or adequate social networks to assist. Allied health professionals highlighted the benefits of a volunteer transport service which was of assistance to improve access but was not available to all.

*“We have issues with transport at our repat campus. We have volunteer transport at [urban_health_service], which makes it a lot easier for us for clients to access our services.”*

ID10_Urban_PT_F_51+

Regardless of compensation status, for people in regional areas, there was limited availability of taxi services, attendant carers and public transport. Thus, even if funding was available for taxis, access to health services was restricted as no taxi service appropriate to their needs existed.

*“I’ve got a client in [regional town] … [Injury_Insurer_2] will say we’ll fund you a taxi transport to get to your appointment. Well, there is no taxi there …”*

* ID14_Statewide_Other_F_37–43

## 4. Discussion

This study provides insight into factors impacting access to care for people following serious injury. Many AHPs perceived that complex funding systems and health services restrict access in both urban and regional areas. Limited availability of necessary AHPs was consistently reported, which particularly restricted access to mental healthcare. Access to healthcare was also felt to be hindered by a reliance on others for transportation as well as the cost, emotional stress and often lengthy time of travel, especially for those living in regional areas.

Our results support the model of access to healthcare conceptualised by Levesque and colleagues which includes accessibility from the health service side as well as factors pertaining to the abilities of the healthcare seeker to utilise the health service [9]. The issues of “approachability”, “availability”, “affordability” and “appropriateness” of a service, in addition to the individual being able to perceive healthcare needs, and reach, pay and engage with a health service were demonstrated by the themes raised by participants. The dimension of ‘acceptability’ pertaining to access varying due to the values of an individual, culture and social norms was one area where our findings varied from the Levesque framework. However, without interviewing the injured individuals themselves, it is hard to gauge whether this issue also impacts access to healthcare in this population.

Previous research has highlighted that the health literacy environment, that is, the knowledge of health professionals, infrastructure, policies and processes, impact how accessible health services are to individuals [8,10,29,30]. Both urban- and regional-based allied health professionals in this study similarly perceived that challenges in navigating complex systems were detrimental to the accessibility of a health service for injured individuals. The findings of this study complement previous research on the NDIS, which suggested that AHPs and people with disabilities perceived rigid funding structures and difficulty accessing support as limitations to accessing necessary care [17,31]. Making systems more user friendly, introducing health information and policies in plain language and working with consumers to optimise technological and social media advances, may assist individuals and health professionals to access information and health services [8,10,29].

Mental health disorders are highly prevalent and can be disabling for many years following serious injury [5], impacting on an individual’s ability to engage in rehabilitation and life roles [8]. In this study, AHPs perceived a shortage of available services in regional areas and out-of-pocket expenses associated with treatment, in urban and regional settings, as key barriers to accessing mental health services. Mental health services in Australia have long been considered as ‘urban centric’, resulting in limited access for those living in regional areas [32] and a higher unmet service need compared to urban areas [2]. Limited mental health services in regional areas have also been reported to compromise patient care and result in AHPs without sufficient expertise being required to provide psychological support [8]. Telehealth systems can provide an alternative service delivery method to improve the reach of psychological services in regional areas where skills shortages exist [33,34]. Given the large number of people at risk of financial hardship following injury [35], greater importance should be placed on ensuring mental health support services regardless of geographic location can be easily accessed and bulk-billed for people following serious injury. Improving the availability and subsequent access to mental health services in regional areas must be a health priority.

For people living in regional areas, physical barriers to accessing healthcare, including transport needs, costs and limited service availability, have been well documented [4,8,36]. Similarly, we found these issues relating to the physical inability to reach a service were barriers to access in both urban and regional areas. One reason for this affecting metropolitan Melbourne may be the large urban sprawl [37]. With more are people living in ‘outer urban’ areas also having to travel significant distances and durations to health services, this may be a barrier to accessing specialised care in inner metropolitan areas. The distribution and growth of urban areas must be considered as a factor in planning for new health services or satellite sites to optimise access to healthcare [37].

Similar to our findings, Christie et al. highlighted the important role of social networks to support transport needs of people following serious injury [7]. Furthermore, they reported that people who were reliant on public transport services had reduced access to services which was perceived to hinder recovery [7]. Our results suggest that, for many receiving compensation following injury, the costs of taxi transport to health services may not a barrier if there are taxis available in their community. To reduce reliance on social networks and improve accessibility for those in urban areas without funded transport options, reduced taxi fare schemes or partnering with one of the many rideshare organisations to enable discounted travel may improve access to healthcare.

Incorporating technology in healthcare provides greater opportunity to connect individuals with necessary services [29]. We found that AHPs across urban and regional settings repeatedly suggested the use of telehealth to improve access to health services, but noted barriers such as technology and cultural resistance to the limited uptake. Due to the coronavirus (COVID-19) pandemic, the use of telehealth for medical and allied health consults has dramatically increased [38]. Supporting the findings of our study, the necessity for implementation has resulted in an increased awareness by clinicians of the advantages of telehealth such as service flexibility and reduced travel burden for patients [38]. The use of telehealth has also grown due to government policy incentivising telehealth consultations, particularly for general practitioners (GPs), encouraging them to reconsider how they practice [39]. The changes to GP and primary care practices have been so marked that early reports suggest that, despite early skepticism from medical professionals, telehealth practice has improved the number of people doctors can review each day [40]. With a more widespread uptake of telehealth services throughout COVID-19 pandemic, the concerns around digital literacy skills, infrastructure development and ongoing feasibility telehealth are now better understood [41,42]. For these learnings to be sustainable beyond the pandemic long term, it will be necessary to have ongoing support from funding bodies and telehealth to be integrated within the general model of care rather than a standalone service [43,44]. The progress made in telehealth through this time can be translated to other cohorts, including seriously injured people, to improve access to care and reduce the physical, mental and financial stress of travel for those with capacity to engage in such services [29,42].

The management of people following serious injuries is commonly multidisciplinary. Due to the complex nature of managing these patients with multiple system injuries, the expertise of a wide variety of AHPs post-hospital discharge is necessary to optimise recovery. The inclusion of a variety of AHPs from different background is a strength of this study by providing a breadth of experiences and perceptions. Given the often-chronic nature of serious injury and long-term involvement with allied health professionals, a number of issues raised by participants in this study are also likely to be relevant other chronic health conditions who require long-term input by allied health professionals both in Australia and internationally.

Whilst the sample size may be perceived as small, it is appropriate for a qualitative study with a focused approach [45,46]. Other qualitative studies exploring experiences of groups in urban and regional areas have used similar sample sizes [8,47,48]. Furthermore, as the aim of qualitative research is to provide insights and understanding into people’s experiences, the findings are not designed to be generalizable [49]. While the results are specific to the sample population, the insights gained from this study will be useful for clinicians and policy makers who work with people with injuries across urban and regional areas in similar health systems. Further research would assist in providing additional evidence of the factors affecting access to post-discharge health services described in this study to enable meta-synthesis and more definitive conclusions and directions for policy and practice.

Participants in this study treated a range of “seriously injured” people, with varying injury severities. Whilst this may be seen as a limitation, it reflects the true heterogeneity that exists within the trauma population and the challenges associated with managing such diverse conditions. This research was only able to capture a subgroup of AHPs and did not include the experiences of psychologists or speech pathologists who are often involved in the long-term care of this population. However, the neuropsychologists did speak to a number of issues around psychology input more globally. Nor did we include nurses or medical professionals, people with serious injuries or caregivers in this study. Further research may be warranted to explore the perspectives of these additional groups. In Victoria, less than one percent of the population reside in ‘Remote’ and ‘Very Remote’ regions [15]. As a result of this, it was not possible to recruit from these areas and barriers identified pertaining to the regional group in this study may be exacerbated for more rural Australian communities. Finally, although this study focused on AHPs within Victoria, Australia, the findings are likely to be applicable to other jurisdictions with similar trauma system design and funding structures for people following serious injury.

## 5. Conclusions

This study has identified that AHPs perceive that barriers to accessing healthcare following serious injury exist for people in urban and regional areas. The disparity in access to care is particularly prevalent in regional areas where limited availability of AHPs results in significant delays and wait periods. Insufficient access to mental health services is a widespread issue across urban and regional areas which may have dire consequences for patients’ long-term recovery. In regional areas specifically, improving the availability of mental health services must be prioritised to ensure necessary care and support can be provided for patients beyond hospital discharge. Telehealth services are likely to be beneficial to reduce physical discomfort and financial costs associated with prolonged travel to access healthcare and improve accessibility to limited services, such as mental health supports. Additional research is required to better understand how changes to health service delivery models, including the use of technology and telehealth, can improve equitable access to healthcare for people following serious injuries to optimise long-term recovery.

## Figures and Tables

**Table 1:** Strategies implemented for qualitative rigour and trustworthiness.

Strategy	Method of Implementation
Descriptive validity	The interviews were listened to and transcripts read and referred to multiple times. The initial five transcripts were coded by two investigators (J.K. and S.C.B.) to develop the coding framework [25].
Negative case analysis	Any cases that were considered outliers were discussed by the authors [26].
Peer review	Presentation and feedback from peers/colleagues. Authors regularly met to discuss the research design, develop the thematic framework and interpret the analysis [27].
Researcher triangulation	Researcher triangulation involved the use of two researchers in the coding and analysis phases of the research. Authors met weekly to discuss the development of the coding framework, themes, interpretation of the results and recommendations developed from this research [28].
Researcher subjectivity	Researcher positionality discussed to identify potential bias, reflection following each interview and early ideas about a coding framework. Further reflection and documentation continued through development and reporting of findings [28].

**Table 2:** Demographic profile of participants (*n* = 25).

Characteristic		*n* (%)
Age, years (mean (SD))	36.2 (8.5)	
Gender	Male	8 (32)
	Female	17 (68)
Profession	Physiotherapist	13 (52)
	Occupational therapist	5 (20)
	Exercise physiologist	4 (16)
	Other ^a^	3 (12)
Years of clinical experience	1–4	4 (16)
	5–9	9 (36)
	10–14	4 (16)
	15+	7 (28)
Healthcare setting	Hospital outpatients	16 (64)
	Community based	6 (24)
	Private practice	3 (12)

^a^ Other allied health = social worker; neuropsychologist. SD, standard deviation.

## Appendix A. Interview Topic Guide

### Appendix A.1. Participant Attributes

Bulleted lists look like this:

- Age;
- Gender;
- Profession;
- Job title (manager/senior/junior);
- Years of experience as a health professional;
- Years of experience in current role;
- Public or private setting;
- Treatment of compensable patients, yes/no;
- Geographic remoteness region;
- Postcode of health facility.

### Appendix A.2. Introduction

I would like to start by asking you about your role and the type of injured patients you treat. To clarify, this interview will be focusing on the management of patients that have been injured, requiring a non-elective hospitalization because of injury. Injury can include intentional and unintentional causes for example road traffic accidents, falls, gunshot wounds.

### Appendix A.3. The Injured Patients

- What are your experiences with treating trauma patient’s post-hospital discharge? Probe: Have you experienced any particularly complex or difficult patients?
- What type/s of seriously injured patients do you treat? Probe: multiple orthopaedic trauma; TBI; SCI; falls.

### Appendix A.4. Treatment of Injuries

- Can you tell me about the types of treatment and services that you offer to seriously injured patients? How do you feel about providing care to seriously injured patients?
- What information or advice did you receive about your patient’s injury? Is there any other information would you have liked to receive and from who?
- What supports are available to you when you need advice on or assistance with treating a seriously injured patient?
- Do you feel that the service you provide are able to meet the needs of your patients? How could they be improved?
- What other services do you feel are required in your local area to better meet the needs of injured patients?

### Appendix A.5. Resources

- What resources are available to you when caring for seriously injured patients?
- What additional resources do you feel would enable you improve the care of seriously injured patients, if any?
- Can you tell me about your experiences in receiving support from more experienced clinicians or a time you have provided support for more isolated services providers?

### Appendix A.6. Experience with Funding Bodies

- Do you provide care for seriously injured patients who are funded by injury insurers for their injury?
- Can you tell us about your experiences with compensable funding bodies?
- Have your experiences with these injury insurers changed over time?
- Is there anything you feel could be done differently by injury insurers to help your patient’s recovery?
- Is there anything the injury insurers could do differently to work effectively with service providers?

### Appendix A.7. General

- How do you feel about being able to ensure adequate continuity of care between acute hospitals and your service?
- Can you tell me about your experiences with other services or policies relating to optimising different aspects of your patients care? For example, health, transportation, disability or rehabilitation for your patients?
- What else can you tell me about trauma care in your community, specific issues you face, and barriers or facilitators to optimal care for seriously injured patients?

### Appendix A.8. Concluding

- Do you have anything else you would like to add or is there anything that I have not covered that you thought I should?
- Do you have any questions about what we have discussed?

## Appendix B. Additional Supporting Quotes

### Appendix B.1. Administrative Delays and Complex Systems Impede Access to Healthcare

“… unfortunately, the most common scenario is that the referral from the metro hospital hasn’t been activated, so whether it’s been lost or not sent through in a timely way. So they’ve been discharged from hospital, sent back to [regional town] and then no follow-up rehab has happened… we would see those guys potentially four, five, unfortunately sometimes six months post-accident. Probably not six months, maybe more like four months. It’s more common to get that than a direct referral from a metro hospital in the orthopaedic and pain rehab clinics especially. That’s not so the case for spinal clinic, the referrals for spinal tend to be a little bit more successful in getting from metro to Bendigo.” ID17, Regional, EP, M, 30–36

“… making things a little bit more kind of user-friendly so the patients can actually access what they want. I think that’s sort of part of the benefit to the NDIS is having a bit more of that decision-making, like empowering the patient, but if the system is too complicated for them to be able to navigate, then it just puts that same barrier backup that the NDIS is trying to address.” ID2, Regional, EP, M, 23–29

“Claims with [Injury Insurer 1] … they still require a lot more information than what [Injury Insurer 2] does have, there’s a lot more report writing … giving them every inch or millimetre of information to get things approved.” ID8, Regional, OT, F, 44–51

### Appendix B.2. Availability of Appropriate Services Affects Access to Healthcare

“Because we have a lot of clients in the community who live in a town, they might have huge personal care needs, but those needs aren’t being met because there’s not enough carers in the town that they live in. So they’re missing out on services that way, or there’s not enough therapists in that town, or their therapists that don’t have the level of expertise that they might need for their really complex therapy needs. So it is a challenge for sure.” ID12, Statewide, PT, F, 30–36

“… that’s a real key, that if clients were provided with the emotional support and the psychological support right from the word go, and that ability for them to be able to accept their injury, and work with the allied health professionals, then they would have much more positive outcomes…I think the key things are the people that have had traumatic events need to be provided with services to deal with their psychological and emotional health, which isn’t being done effectively at the moment. Their care services need to be improved in this area.” ID8, Regional OT, F, 44–51

“Psychology would also be really helpful for the team, but we, as I said, just have the neuropsyche, and largely assessment based. At the moment psychology just has to be through GP, getting onto a (mental health) care plan. There sort of used to be a tiny bit of access we could get through the hospital for people who had been seen by hospital psychologists. But it’s pretty little access now.” ID6, Urban, PT, F, 22–29

“There’s a real gap with social work services in our community, and even access in psychology. Because even the Medicare rebate in psychology is out of reach for a lot of people. Navigating mental health plans, but also then there is still a huge gap, like you’re talking 60, 80 a hundred dollar gap for each psychology appointment. That’s not achievable for a lot of people.” ID7, Regional, Other, F, 30–36

### Appendix B.3. Physical Ability to Reach Services Can Restrict Healthcare Utilisation

“Transportation is a real issue for a lot of people. And that has a flow-on effect for things like being able to attend therapy appointments. A lot of small towns don’t have maxi taxis, and all of our clients need maxi taxis to get around, if they don’t have their own wheelchair accessible vans and things like that. So it is really challenging for a lot of the rural and regional clients.” ID12, Statewide, PT, F, 30–36

I’ve got a client in [regional town] … [Injury Insurer 2] will say we’ll fund you a taxi transport to get to your appointment. Well, there is no taxi there. Everything’s just much harder for them. They can’t just jump in a taxi or a train, or whatever, to get to see a doctor because firstly, they’re three hours away, and they don’t have taxis out in these areas sometimes.” ID14, Statewide, Other, F, 37–43

“There’s problems when the patients go back to the acute hospitals, to the big trauma hospitals in the city, for reviews and plans and all that kind of thing, and probably 50 per cent of the time the communication back from there is not very good. And so that leads to anxiety and concern about what’s going on. And a lot of people from the country don’t like going up to the big city, or the big city hospitals, they find it really stressful and annoying …” ID3, Urban, Other, F, 37–43
